# Electrical Circuits That Supply Constant Electric Fields *In Vitro*

**DOI:** 10.1089/bioe.2019.0036

**Published:** 2020-09-16

**Authors:** Masayuki Yamashita

**Affiliations:** Center for Basic Medical Research, International University of Health and Welfare, Ohtawara, Japan.

**Keywords:** galvanotropism, electric field, field strength, voltage difference, feedback circuit

## Abstract

***Background:*** To study the molecular mechanism of galvanotropism *in vitro*, the strength of an electric field (EF) must be controlled precisely. In this study, I present a culture system that supplies an EF of constant strength by regulating the amount of current supplied to the culture medium.

***Materials and Methods:*** Voltage of the medium was recorded at two fixed points along the current flow throughout the culture period. The voltage drop between the two points was maintained at the desired value by a negative feedback circuit and an operational amplifier.

***Results:*** The field strength was defined by the voltage drop and the distance between the two points, and in this system, the EF can range from 0.0005 to 15 mV/mm.

***Conclusions:*** This culture system may be a useful tool to determine the nature of galvanotropism.

## Introduction

Galvanotropic cell behavior has been studied in tissue culture and dissociated cells with electrical current applied to culture medium from a direct current (DC) power supply.^1^ The first report on the effect of electricity on axon growth was published in 1920 soon after nervous system cells were able to be cultured *in vitro*.^2,3^ It was believed that the direction of axon growth was determined by the electric field (EF) around the axons.^4^ This idea has been termed “galvanotropism” because Galvani discovered animal electricity in frog nerves in 1791.^5^

Many questions regarding the molecular mechanisms of galvanotropic cell behavior remain. When studying the effects of electricity on cells, the strength of the EF must be described quantitatively and the field strength around the cell should be controlled precisely.

The strength of the EF depends on the current flow in the culture chamber, and the amount of current varies with factors, such as resistance of the culture medium, temperature, viscosity, and presence of ionic components. The resistance of the junction between the electrode supplying the current and the culture medium affects the amount of current applied, and these factors can change over time. Thus, it is difficult to provide a constant EF using conventional, commercially available constant voltage or current sources. The shape of the culture chamber also affects field strength because current density depends on the path of the current. Recently, a method to generate a uniform EF using circular-shaped culture plates with polymeric inserts has been developed.^6^

This study describes an electrically controlled culture system that provides a uniform constant DC EF of desired strength. The EF is formed in a long narrow trough filled with culture medium, and electrical currents flow along the long axis of the trough. Two electrodes are inserted into the culture medium at two fixed points along the current flow. The voltage drop between the two points is continuously monitored, and a negative feedback circuit adjusts the voltage drop to the desired value by regulating the amount of current applied. This system can provide a constant EF from 0.0005 to 15 mV/mm and is suitable for studying the effects of weak EFs.

A study on galvanotropic axon orientation using this device has been published,^7^ and the method section of this previous study is reproduced here with new figures to improve the description of the culture system. Previous studies used conventional galvanotaxis chambers in which voltage differences were not constantly recorded.^8–10^ However, one galvanotaxis study used a visual feedback system.^11^

## The Culture Chamber

**FIG. 1.**
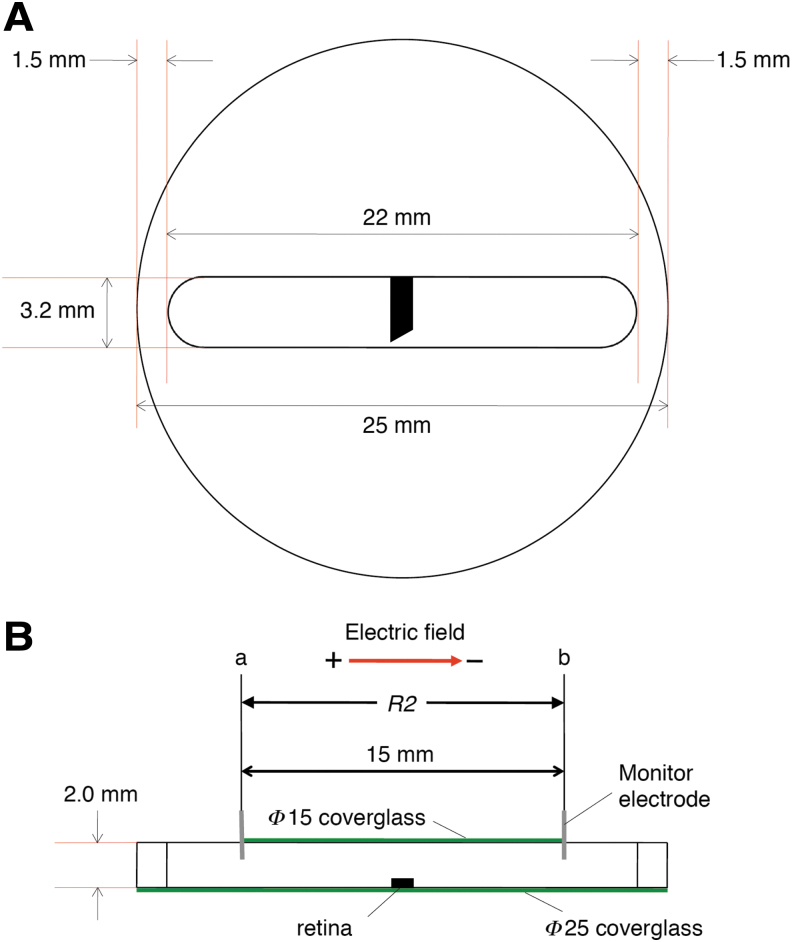
Culture chamber made from an acrylic disk. Retinal tissue was placed in the middle of the trough filled with culture medium. **(A)** Top view. **(B)** Side view. Voltage was recorded at two points (a and b).

The culture chamber can be made from a thin acrylic disk or an acrylic plate of any form. First, a long trough was formed in the acrylic disk (Φ25) or plate (Fig. 1A). A cover glass the same size as the acrylic disk was secured to the bottom of the trough using silicone grease (Dow Corning, H.V.G). Cells or tissues were placed on the bottom middle of the trough. To regulate the volume of culture medium in the trough, another round cover glass (Φ15) was secured to the top of trough with silicone grease (Fig. 1B). Then, the trough was filled with 100 μL of culture medium.

**FIG. 2.**
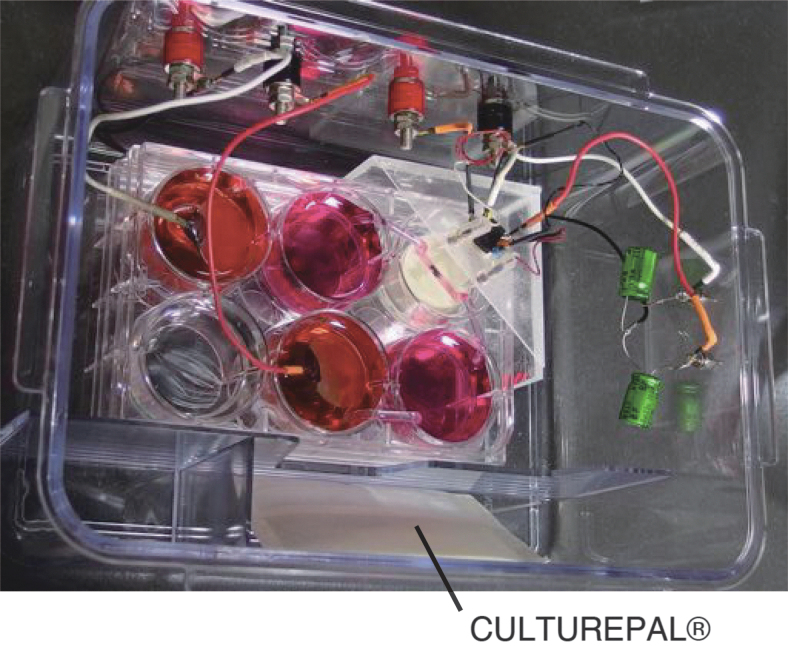
The inside of an airtight jar containing the six-well culture plate and CULTUREPAL^®^.

A conventional six-well culture plate was used to make the entire electrical circuit. Because the culture chamber was too thin to place into a well of the culture plate, the culture chamber was put on an acrylic disk (Φ30, 10 mm height), and paper of the same size as the culture chamber was inserted between the bottom of the chamber and the acrylic disk to easily separate them after the incubation period. Then, the culture chamber and the acrylic disk were placed in a corner well of the six-well culture plate (Fig. 2). To maintain humidity, 1 mL of distilled water (DW) was injected around the base of the acrylic disk.

## The Current Application Apparatus

Two U-shaped glass tubes, which were made from Φ3 (outer diameter) glass tubes, were completely filled with culture medium, and inserted into the trough at both ends. If air bubbles entered the glass tube, current flow was interrupted. The other end of each tube was inserted into the neighboring well, which was filled with an excess volume (14 mL) of culture medium to prevent changes in the composition of the medium around the cell due to electrophoresis.

**FIG. 3.**
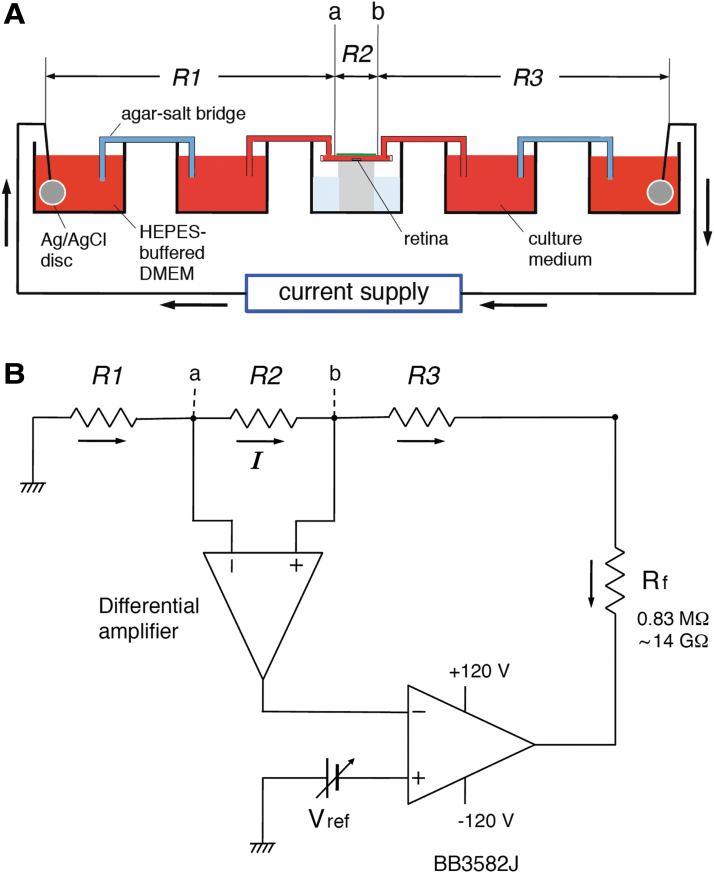
The electrical current flow and negative feedback circuit. **(A)** Current flow through the chamber. **(B)** Diagram of the negative feedback circuit. Rf was decreased to ≤0.83 MΩ for an EF of ≥10 mV/mm. EF, electric field; Rf, feedback resistor. Voltage was recorded at two points (a and b).

The two wells filled with culture medium were connected to the neighboring two wells, which were filled with Dulbecco's modified Eagle's medium (DMEM) buffered with 25 mM HEPES. The anode and cathode electrodes were inserted into the wells containing the HEPES-buffered solution to supply the current. To avoid diffusion between the two solutions (culture medium and HEPES-buffered solution), agar-salt bridges, which were prepared by filling a U-shaped glass tube with 1% agar in saline, were inserted into the two wells on the anode and cathode sides (Figs. 2 and 3A). Phenol red was used to monitor the pH in each well. A total of five wells were used to make the entire electrical circuit, and the remaining well was filled with DW to maintain humidity.

After filling the six wells, the culture plate was placed into a 2.5 L airtight jar containing CULTUREPAL^®^ (Mitsubishi Gas Chemical Company, Inc., Tokyo). CULTUREPAL provides 5% CO_2_ within an airtight jar for up to 7 days (Fig. 2). Because humidity and CO_2_ were maintained within the airtight jar, a conventional CO_2_ incubator was not used. The airtight jar was placed into a 37–38°C dry incubator with a hole for electric cables to pass through.

## Electrodes for Supplying Current and Monitoring Voltage

Two anode and cathode Ag/AgCl electrodes were placed into the two wells filled with HEPES-buffered DMEM. The size and shape of the Ag/AgCl electrode (up to Φ25) was selected based on the amount of current applied. The electrode for monitoring voltages in the culture medium was made from a Φ3 glass tube by heating it over a small fire and pulling it until the tip diameter became ∼0.3 mm. The tip and the cut edge of the glass pipette were fire-polished, and then the glass pipette was filled with 1% agar in saline. A small Ag/AgCl pellet (Φ1) containing an Ag/AgCl wire was inserted into the glass pipette. The Ag/AgCl wire was soldered to a copper lead wire, and this junction was covered with epoxy resin to prevent contact with the agar-salt gel.

The lead wire was connected to the input of a voltage follower. An LMC662CN amplifier was used for the voltage follower because the input resistance was >1 TeraΩ and the bias current was 2 fA. To minimize the length of the lead wire connected to the monitor electrode and the voltage follower, the LMC662CN was placed near the recording chamber in the airtight jar (Fig. 2). The cables to the anode and cathode electrodes, the outputs of the two voltage followers, and the power supply to the LMC662CN were connected to connectors fixed onto the wall of the airtight jar with silicone grease (Fig. 2).

## The Entire Electrical Circuit with Negative Feedback

The two monitor electrodes were inserted into the culture medium at both edges of the top cover glass (Fig. 1B-a, b). Because the diameter of the cover glass was 15 mm, the distance between the two monitor electrodes was 15 mm. The entire electrical circuit is shown in Figure 3A. *R*2 represents the resistance between the two monitor electrodes (Fig. 3A-a, b). The resistances of the anode and cathode electrodes, the agar-salt bridges, the glass tubes containing the culture medium, and the solutions connected to them are represented by *R*1 and *R*3.

The voltage drop between the two points (a, b) was continuously monitored by the differential amplifier shown in Figure 3B. The output of this differential amplifier was connected to the inverting input (−) of an operational amplifier. BB3582J was used as the operational amplifier because of its wide operating voltage range (113 V). Vref represents the reference voltage used to regulate the voltage drop between the two points by negative feedback. Vref was connected to the noninverting input (+) of BB3582J. *I* represents the feedback current that flows through *R*1–*R*3 and the feedback resistor (Rf). The output of the differential amplifier equaled Vref upon negative feedback by the operational amplifier.

**FIG. 4.**
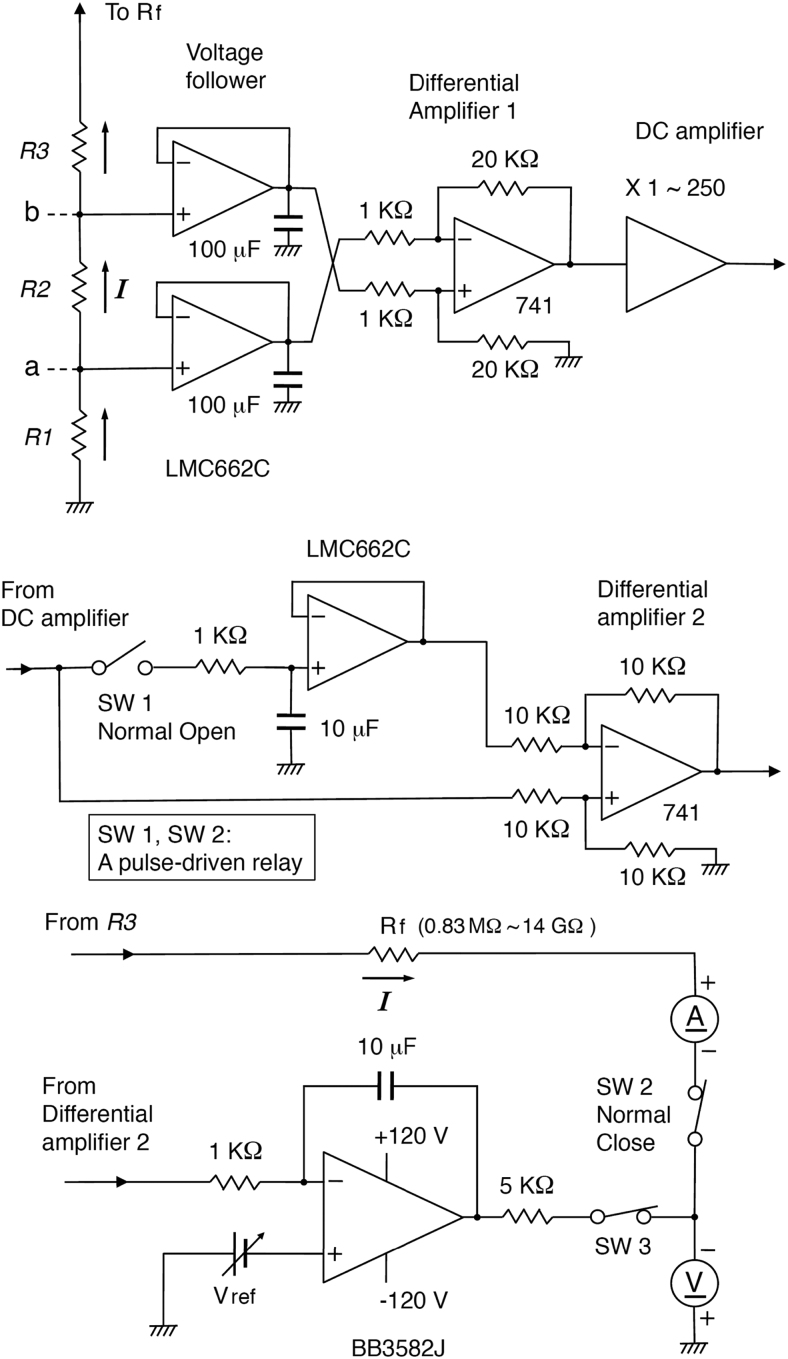
The details of the entire electrical circuit. The two resistors in differential amplifier 1 (20 KΩ) and Rf were reduced for EFs ≥10 mV/mm.

**FIG. 5.**
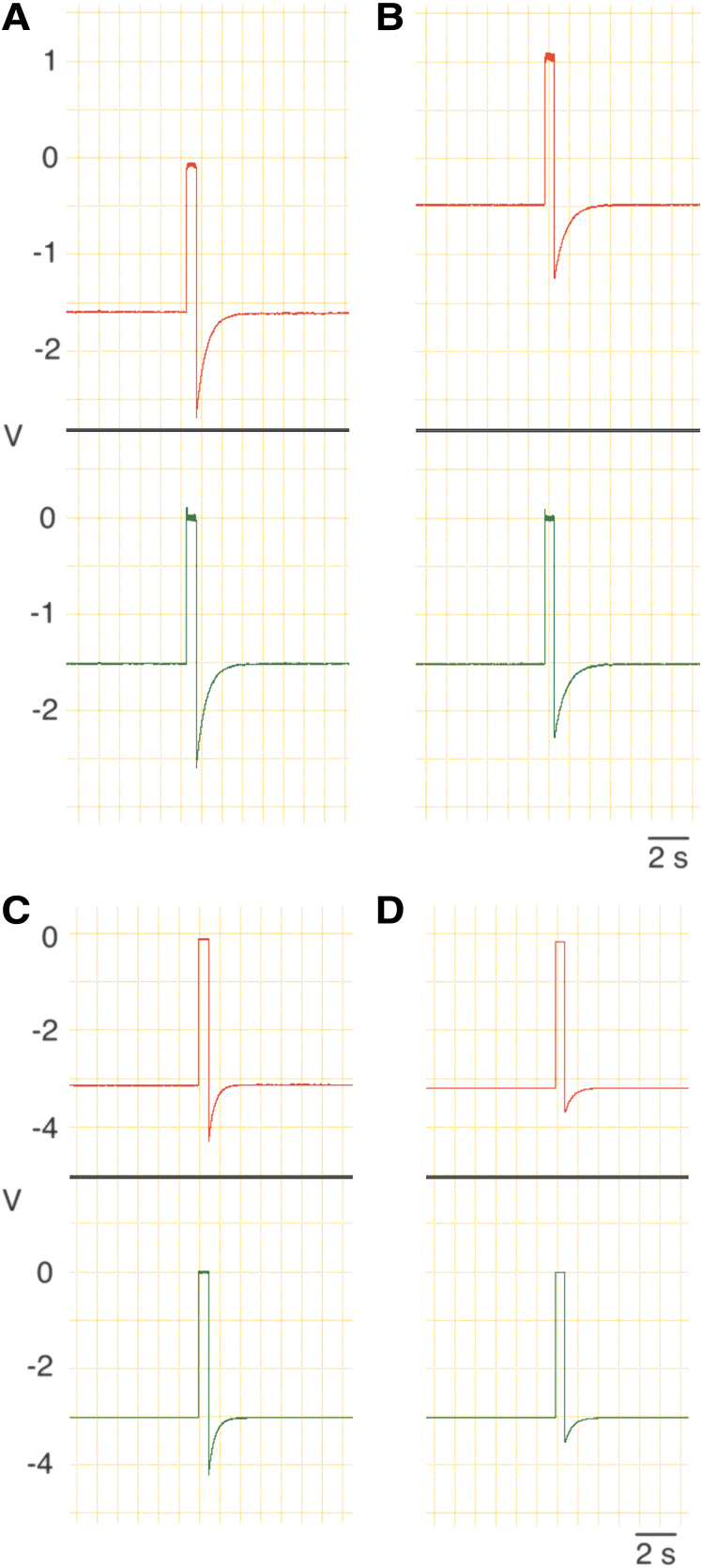
Outputs of the DC amplifier (red) and differential amplifier 2 (green). **(A, B)** An EF of 0.1 mV/mm was supplied. The voltage difference between the two monitor electrodes (distance, 15 mm) was amplified 1,000-fold. Vref, −1.5 V; Rf, 70 MΩ. **(A)** Records taken at the beginning of the culture. **(B)** Records taken after 21 h of culture. Note that the output of the DC amplifier changed positively, whereas the output of differential amplifier 2 did not change upon subtraction of the offset voltage. **(C, D)** An EF of 2.0 mV/mm was supplied. The voltage difference between the two monitor electrodes was amplified 100-fold. Vref, −3.0 V; Rf, 4.3 MΩ. **(C)** Records taken at the beginning of the culture. **(D)** Records taken after 23 h of culture. Sampling frequency, 100 Hz. DC, direct current; Vref, reference voltage.

The offset voltage of the differential amplifier, including offsets of the two monitor electrodes and the two voltage followers, was nullified by subtracting the output voltage recorded during current-off (*I* = 0) periods of 0.5 s at an interval of 100 s (duty cycle, 99.5%). The current flow was interrupted with a pulse-driven relay switch, which opens SW 2 and closes SW 1 during the current-off period (Fig. 4). The outputs of the DC amplifier and differential amplifier 2 were continuously recorded with a 2-channel chart recorder (PowerLab 2/26; ADInstruments) throughout the culture period to check that all components are operating properly (Fig. 5).

The output of the DC amplifier was connected to a low-pass filter (cutoff frequency, 500 Hz) to eliminate high-frequency noises. Hum Bug (Quest Scientific, North Vancouver, Canada) was used to eliminate line noises when weak EFs (≤0.1 mV/mm) were used. The minimum limit for the EF strength depends on the signal-to-noise ratio. The voltage drop between the two points was detectable at an EF of 0.0005 mV/mm (Rf, 14 GΩ; total gain, 5,000; Vref, −37.5 mV; *I* = about 5 nA).

## Application

The culture system presented here was used to study galvanotropic behavior of retinal ganglion cell axons. Retinal strips from chick embryos were embedded in Matrigel^®^ and cultured in a constant DC EF of 15 mV/mm to mimic the *in vivo* EF.^12^ The current supplied increased from 150 to 200 μA for 24 h. The monitor electrodes were prepared just before the retinal strip was prepared. The electrodes were cleaned in boiling water after each culture. The details of the retinal strip culture have been described previously.^13^

## Summary

The culture system presented in this study establishes a reliable method for supplying a constant EF to study the molecular mechanisms mediating galvanotropic cell behaviors *in vitro*. Galvanotropic effects can be observed at various field strengths ranging from 0.0005 mV/mm to >100 mV/mm.^1,7–11^ This culture system, which is based on a feedback circuit, may be a useful tool to determine the nature of galvanotropism.
